# Preparation of Hydrophobic Au Catalyst and Application in One-Step Oxidative Esterification of Methacrolein to Methyl Methacrylate

**DOI:** 10.3390/molecules29081854

**Published:** 2024-04-19

**Authors:** Yanxia Zheng, Yubo Yang, Yixuan Li, Lu Cai, Xuanjiao Zhao, Bing Xue, Yuchao Li, Jiutao An, Jialiang Zhang

**Affiliations:** 1 Institute of Clean Chemical Technology, School of Chemistry and Chemical Engineering, Shandong Collegial Engineering Research Center of Novel Rare Earth Catalysis Materials, Shandong University of Technology, Zibo 255049, China; yanxia2020@126.com (Y.Z.);; 2 School of Mechanical Engineering, Shandong University of Technology, Zibo 255049, China; 3 School of Resources and Environmental Engineering, Shandong University of Technology, Zibo 255049, China; 4 Shandong Mingsheng Environmental Protection Technology Co., Ltd., Jinan 250000, China

**Keywords:** hydrophobic catalysts, methyl methacrylate, oxidative esterification, Au catalyst

## Abstract

The water produced during the oxidative esterification reaction occupies the active sites and reduces the activity of the catalyst. In order to reduce the influence of water on the reaction system, a hydrophobic catalyst was prepared for the one-step oxidative esterification of methylacrolein (MAL) and methanol. The catalyst was synthesized by loading the active component Au onto ZnO using the deposition–precipitation method, followed by constructing the silicon shell on Au/ZnO using tetraethoxysilane (TEOS) to introduce hydrophobic groups. Trimethylchlorosilane (TMCS) was used as a hydrophobic modification reagent to prepare hydrophobic catalysts, which exhibited a water droplet contact angle of 111.2°. At a temperature of 80 °C, the hydrophobic catalyst achieved a high MMA selectivity of over 95%. The samples were characterized using XRD, N_2_ adsorption, ICP, SEM, TEM, UV-vis, FT-IR, XPS, and water droplet contact angle measurements. Kinetic analysis revealed an activation energy of 22.44 kJ/mol for the hydrophobic catalyst.

## 1. Introduction

Methyl methacrylate (MMA) is a versatile chemical compound widely used in various industries, including the manufacturing of plexiglass, plastics, resins, and coatings. The industrial production of MMA is mainly through the acetone cyanohydrin method (ACH process), the ethylene carbonylation method, and the isobutylene oxidation method [1,2]. However, the former two methods suffer from serious environmental and economic drawbacks, such as the use of highly toxic hydrogen cyanide, the high cost of waste ammonium bisulfate treatment, and the harsh conditions of the transportation and storage of ethylene [3]. The two-step oxidation process of the isobutylene oxidation method includes the oxidation of isobutylene to methacrolein (MAL) and the oxidative esterification of MAL with methanol in an oxygen atmosphere [4,5]. Synthesis of MMA via one-step oxidation and esterification of methacrolein with methanol is a green and sustainable way, which has a high atom utilization rate, no pollution, and environmental friendliness, and has attracted wide attention in recent years [6,7,8,9,10,11,12]. The construction and modification of gold catalysts have shown excellent performance in CO oxidation reactions [13,14], oxidative esterification reactions [15,16,17,18,19,20,21], and other fields. However, the alkaline sites of the support in gold-based catalysts, the particle size of gold nanoparticles, and the interaction between the support and gold nanoparticles all have a certain impact on the reaction performance. The activity and stability of gold-based catalysts still need to be improved. By synthesizing gold nanoparticles with a core–shell structure, doping other metals, or adding additives, the electronic structure of metals can be changed to promote the stability and catalytic performance of gold catalysts [22].

The hydrophobic effect has proven effective in many catalytic fields. Catalytic activity, product selectivity, and catalyst stability are strongly related to catalyst hydrophobicity [23]. The core–shell FeMn@Si catalyst with excellent hydrophobicity was prepared by Ding’s team [24,25], and they used the catalysts for Fischer–Tropsch synthesis (FTS). The hydrophobic shell protected the active site from oxidation using the generated water, restrained the side reactions related to water, and improved CO conversion and olefin yield during the reaction. Xiao’s team [26,27] synthesized hydrophobic catalysts through the fixation of AuPd alloy nanoparticles within aluminosilicate zeolite crystals, followed by modification of the external surface of the zeolite with organosilanes. They used it for syngas conversion and compared the hydrophobic degree of different hydrophobic groups and their influence on the syngas conversion, with methanol selectivity reaching 92%. They discussed chemical modification for hydrophobization of the catalysts, specifically mentioning hydrophobic promoters that could improve syngas conversion, and suggested precisely regulating the wettability of the catalysts. Wang’s team [28,29,30,31,32,33] studied the application of hydrophobic catalysts in oxidative esterification reactions. They prepared a hydrophobic catalyst with a hydrophobic SDB carrier loaded with mono/multimetal and used it for the first time in the one-step oxidative esterification reaction. The SDB-supported catalyst could be reused for long-term cycles without a decrease in activity. Compared with hydrophilic catalysts, hydrophobic catalysts are more active than catalysts supported on hydrophilic materials like γ-Al_2_O_3_ and SiO_2_. It proves that the high activities exhibited by hydrophobic catalysts are directly related to their hydrophobicity. It is necessary to study the hydrophobic catalysts and explore the reaction mechanism for the oxidative esterification reactions.

In this work, we synthesized hydrophobic catalysts by constructing a core–shell structure and grafting hydrophobic organic groups, intending to improve their performance in the oxidative esterification reaction. The resulting catalysts were thoroughly characterized using various techniques, including XRD, BET, TEM, SEM, ICP, XPS, and Water-droplet contact angles. The reaction mechanism and kinetics were investigated.

## 2. Results

### 2.1. XRD Analysis

**Figure 1:**
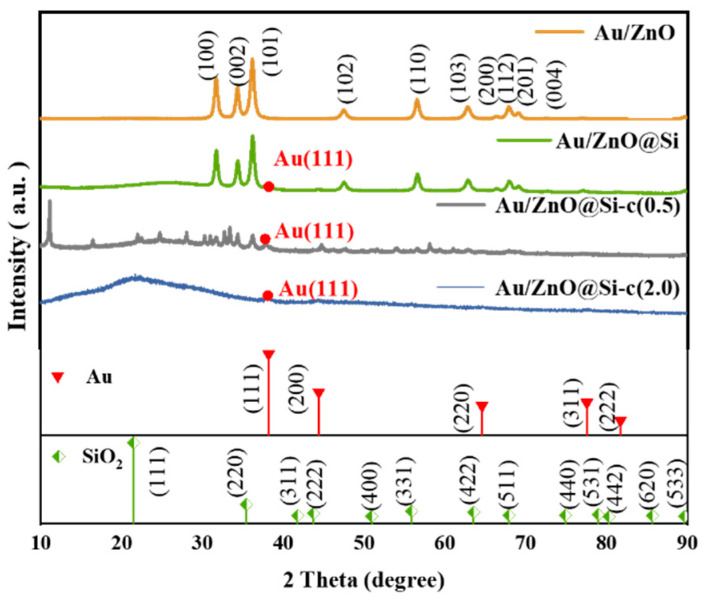
XRD patterns of the catalysts.

The XRD patterns of the catalysts are shown in Figure 1. The intense peaks at 2θ = 31.7°, 34.4°, 36.2°, 47.5°, 56.6°, 62.8°, and 67.9° correspond to the (100), (002), (101), (102), (110), (103), (112), and (201) planes of ZnO, respectively, with lattice parameters consistent with reported data JCPDS file No. 36-1451. The absence of the Au (111) diffraction peak at 38.2° indicates a higher dispersion and smaller particle size of Au NPs on ZnO. After the construction of the silicon shell, the characteristic diffraction peaks of ZnO had no evident change, but the characteristic diffraction peak of Au (111) appeared, indicating that the particle size of Au became larger after the construction of the silicon shell. A bulge at 2θ < 30° may be the diffraction peak of Si. After hydrophobic modification, the diffraction peaks of ZnO were significantly changed; the characteristic diffraction peaks of ZnO were no longer obvious, and the bulge with 2θ < 30° was changed.

### 2.2. SEM and TEM Images of the Catalysts

**Figure 2:**
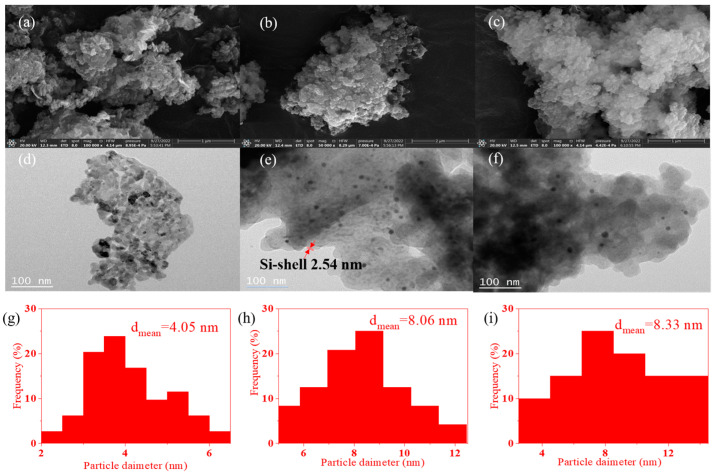
SEM and TEM images of Au/ZnO (**a**,**d**,**g**), Au/ZnO@Si (**b**,**e**,**h**), and Au/ZnO@Si-c(2.0) (**c**,**f**,**i**).

Figure 2 displays SEM, TEM, and Au particle size distribution images of Au/ZnO, Au/ZnO@Si, and Au/ZnO@Si-c(2.0). The particle morphology of ZnO was observed. The SEM image of Au/ZnO@Si reveals a relatively smooth surface profile, indicating the uniform silicon shell coating of Au/ZnO (Figure 2e). Similarly, TEM images of Au/ZnO@Si-c(2.0) confirm that Au/ZnO is encapsulated within an amorphous silicon shell (Figure 2f). As depicted in Figure 2g–i, the average diameter of Au NPs for Au/ZnO, Au/ZnO@Si, and Au/ZnO@Si-c(2.0) is 4.05 nm, 8.06 nm, and 8.33 nm, respectively. This suggests that the gold particles agglomerate and grow larger during the process of silicon shell encapsulation and hydrophobic modification. Furthermore, Figure S2 demonstrates the uniform distribution of Au, Zn, O, Si, and other elements on the catalysts Au/ZnO@Si and Au/ZnO@Si-c(2.0), with silicon forming a sealed shell on the surface.

### 2.3. N_2_ Physisorption

**Figure 3:**
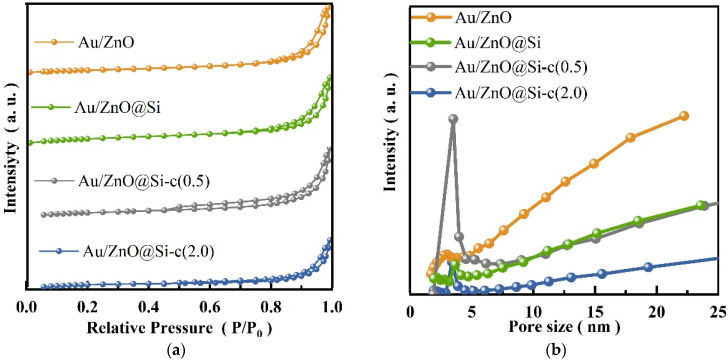
(**a**) Specific surface area diagram of sample; (**b**) BJH pore size distribution of the sample.

Figure 3 displays the N_2_ adsorption–desorption isotherms and pore size distribution of the samples. All isotherms of the samples showed a hysteresis loop categorized as type IV, indicating the presence of mesoporous materials. The encapsulation of SiO_2_ on Au/ZnO results in the appearance of more mesopores with pore sizes ranging from 2 to 50 nm, indicating the presence of mesopores in the SiO_2_ shell.

Table 1 provides the BET surface area and crystalline diameter of the samples. The BET surface area of Au/ZnO was found to be 43.4 m^2^ g^−1^, while the surface areas of the encapsulated samples, Au/ZnO@Si, Au/ZnO@Si-c(0.5), and Au/ZnO@Si-c(2.0), were reduced to 20.1 m^2^ g^−1^, 17.8 m^2^ g^−1^, and 7.6 m^2^ g^−1^, respectively, due to the SiO_2_ encapsulation.

The actual gold loadings measured via ICP-MS were 0.12, 0.1, 0.04, and 0.04 wt% for Au/ZnO, Au/ZnO@Si, Au/ZnO@Si-c(0.5), and Au/ZnO@Si-c(2.0). The treatment of silicon shell encapsulation and hydrophobic modification can lead to the loss of Au.

### 2.4. The CO_2_-TPD of Catalysts

**Figure 4:**
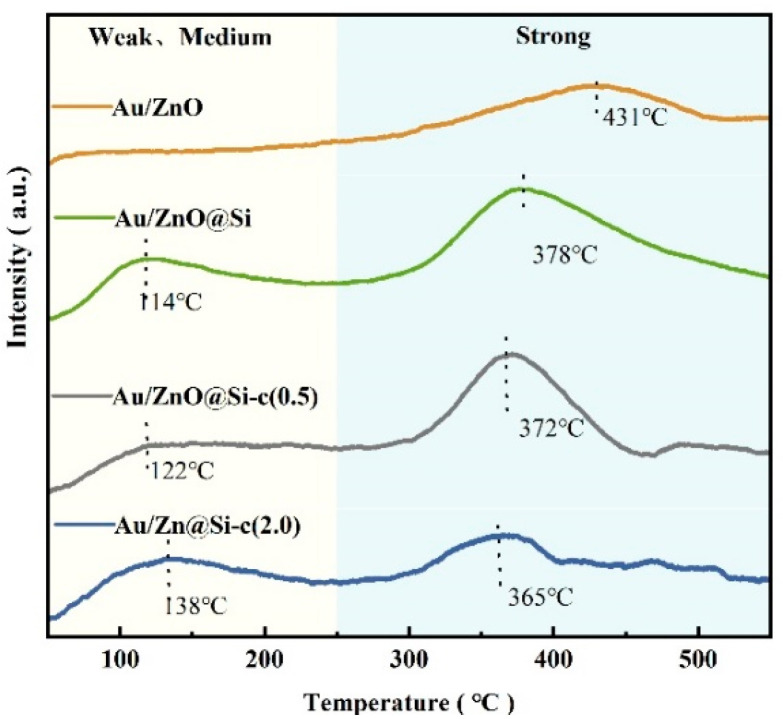
The CO_2_-TPD profiles of the catalysts.

The base properties of the catalysts are shown in Figure 4. The CO_2_ analytical peak around 300 °C–500 °C in the CO_2_-TPD curve is considered the strong basic site, while the CO_2_ analytical peak around 100 °C–150 °C corresponds to the weak basic site. The Au/ZnO catalyst exhibited only a desorption peak around 430 °C ascribed to the strong basic sites. The consumption of CO_2_ during the chemical adsorption is shown in Table 2. The CO_2_ desorption of the high-temperature peak of Au/ZnO was 0.9 CO_2_ mmol per 1 g catalyst. The strong base originates from the basic –OH on the surface of ZnO. The Au/ZnO@Si, Au/ZnO@Si-c(0.5), and Au/ZnO@Si-c(2.0) catalysts presented the weak and medium basic sites around 110 °C–140 °C. After encapsulating the silicon shell, the strong base region of ZnO shifts towards the lower temperature, indicating that the bonding and effect of zinc oxide on carbon dioxide weakened. The CO_2_ desorption of the high-temperature peak of Au/ZnO@Si increased to 2.25 CO_2_ mmol per 1 g catalyst, which may be due to the –OH effect of Si–OH. Furthermore, after hydrophobic modification, the CO_2_ desorption of the high-temperature peak decreased, which may be due to the combination of organic groups and Si–OH, and the decrease of –OH. This indicates that the methyl group is successfully grafted on the surface of the silicon shell.

### 2.5. UV–Vis Characterization of Catalysts

**Figure 5:**
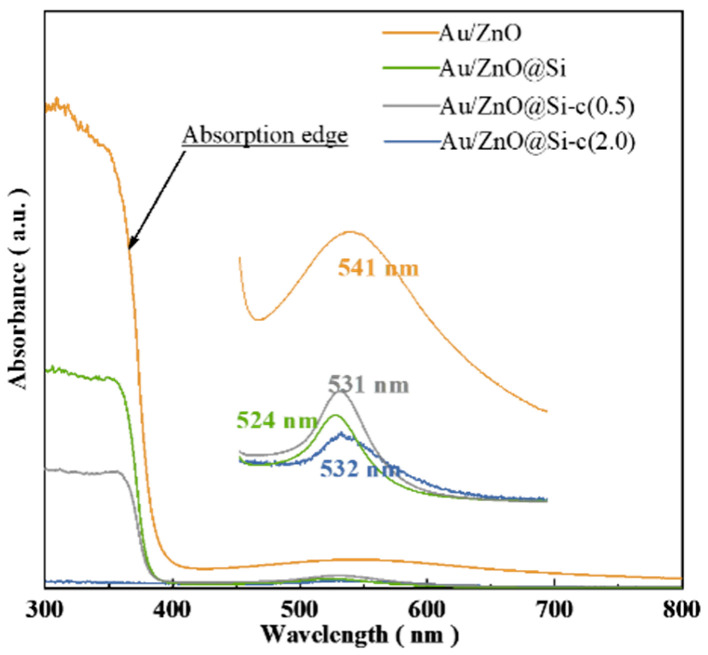
UV–vis spectra of catalysts.

Figure 5 illustrates the UV–vis spectra of Au/ZnO, Au/ZnO@Si, Au/ZnO@Si-c(0.5), and Au/ZnO@Si-c(2.0). In Figure 5, the adsorption edge of ZnO is observed at approximately 380 nm. Upon the construction of the silicon shell, the adsorption of ZnO decreases. Furthermore, the addition of hydrophobic reagents further reduces the adsorption of ZnO. Figure 5 shows the UV–visible spectrum, highlighting the absorption peak of Au. After constructing the silicon shell, the absorption peak of Au undergoes a blue shift, and the absorption amount is significantly reduced. This blue shift may be attributed to the introduction of the silicon hydroxyl group during the construction of the silicon shell.

### 2.6. XPS Analysis

**Figure 6:**
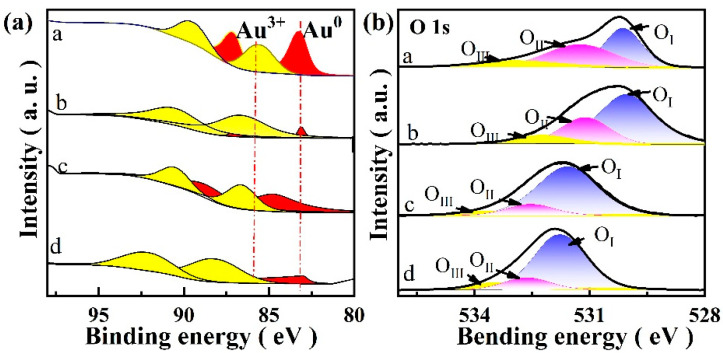
(**a**) Au 4f and (**b**) O 1s XPS spectra of catalysts. a: Au/ZnO; b: Au/ZnO@Si; c: Au/ZnO@Si-c(0.5); d: Au/ZnO@Si-c(2.0).

XPS analysis was conducted on the catalyst, as depicted in Figure 6. The XPS spectrum’s binding can be calibrated with C1s (284.8 eV). Despite the signal overlap between the Au 4f peak and the Zn 3p peak, we convolve the Au 4f peak [34,35,36,37]. XPS spectra reveal that all catalysts exhibit similar Au 4f curves and can be differentiated into Au 4f_7/2_ and Au 4f_5/2_ spin states. The peak positions of different samples, the proportions of Au elements with different chemical valences, and the proportions of Au with different valences in XPS analysis are presented in Table 3. After hydrophobic modification, the peak position of the Au 4f in the Au/ZnO@Si-c(0.5) sample shifts to the higher field, ΔE = 0.9 eV. This phenomenon may be attributed to the chemical binding of the modifier to the sample, which enhances the electron cloud density on the Au and O surfaces [36]. However, metallic Au and reactive oxygen species can effectively promote the oxidative esterification of aldehyde and methanol [38,39].

The peak fitting results corresponding to O 1s are presented in Table S1. Three deconvolution peaks of oxygen were observed, presumed to be surface lattice oxygen (O_I_), adsorbed oxygen (O_II_), and hydroxyl oxygen (O_III_) [40,41]. Components at B.E. = 530.1 eV, B.E. = 532.1 eV, B.E. = 532.3 eV, and B.E. = 531.8 eV are attributed to O_I_, while components at B.E. = 531.3 eV, B.E. = 533.0 eV, B.E. = 533.2 eV, and B.E. = 533.3 eV are attributed to O_II_. The components at B.E. = 532.3 eV, B.E. = 533.8 eV, B.E. = 533.4 eV, and B.E. = 534.3 eV belong to O_III_. The percentage of adsorbed oxygen and lattice oxygen on the surface of the hydrophobic catalyst increased significantly, with the total percentage of adsorbed oxygen and lattice oxygen exceeding 90%. The oxygen on the surfaces of the Au/ZnO@Si-c(0.5) and Au/ZnO@Si-c(2.0) catalysts exhibit symmetric characteristic peaks at 532.3 eV and 532.8 eV, respectively. Before and after hydrophobic modification, the peak positions shift to higher field intensities, with ΔE values of 0.20 eV and 0.70 eV, respectively, indicating a loss of electrons from the surface oxygen element.

### 2.7. FT-IR of the Catalysts

**Figure 7:**
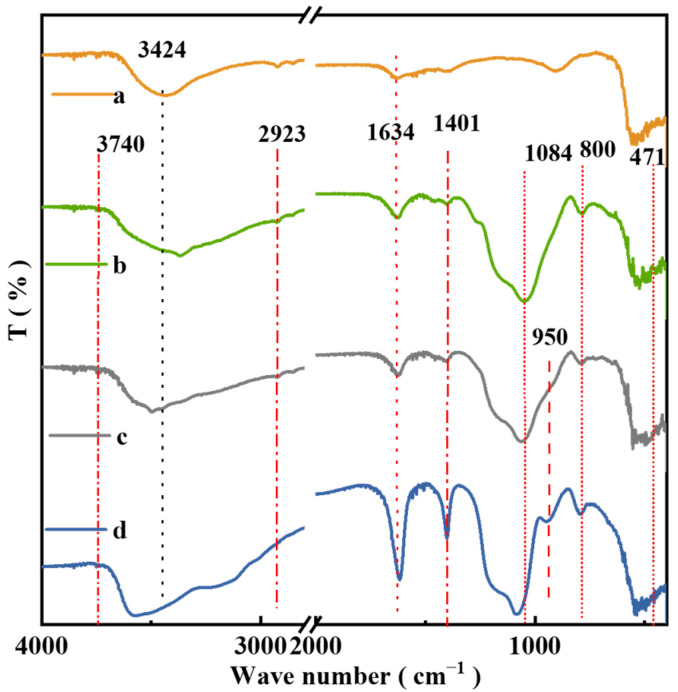
FT-IR spectra: (a) Au/ZnO, (b) Au/ZnO@Si, (c) Au/ZnO@Si-c(0.5), and (d) Au/ZnO@Si-c(2.0).

The FT-IR spectra of Au/ZnO, Au/ZnO@Si, Au/ZnO@Si-c(0.5), and Au/ZnO@Si-c(2.0) are presented in Figure 7. The bands observed at 3424 and 1634 cm^−1^ correspond to the vibration of –OH bonds, while the bands at 471, 800, and 1084 cm^−1^ correspond to the vibration of Si–O–Si bonds in the SiO_2_ shell. Additionally, an absorption band appears at 950 cm^−1^, corresponding to the stretching vibration of Si–OH. The presence of isolated silanol groups was confirmed using the OH stretch at 3740 cm^−1^ on Au/ZnO@Si, suggesting the possibility of introducing hydrophobic –CH_3_ groups through silanization reactions. The bands observed at 2923 cm^−1^ and 1401 cm^−1^ can be attributed to the stretching and bending vibrations of –CH_3_, respectively, confirming the successful modification of organic groups onto the catalyst through post-silylation.

### 2.8. Water-Droplet Contact Angles of the Catalysts

**Figure 8:**
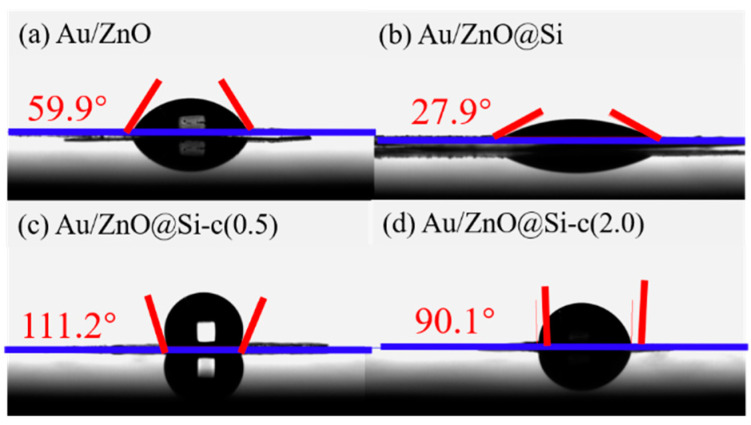
Water-droplet contact angles of various catalysts.

Figure 8 presents the water-droplet contact angles of various catalysts, highlighting the impact of hydrophobic modification on surface properties. The Au/ZnO@Si catalyst exhibits a contact angle of 27.9°, suggesting a hydrophilic surface (Figure 8b). However, after hydrophobic modification through TMCS, the contact angle increases to 111.2° over the Au/ZnO@Si-c(0.5) surface, indicating a complete transformation of the Au/ZnO@Si surface from hydrophilic to hydrophobic. Furthermore, different hydrophobic abilities of Au/ZnO@Si-c catalysts were achieved by varying the TMCS coverage. As shown in Figure 8c,d, increasing the TMCS exposure enhances the water contact angle from 27.9° for Au/ZnO@Si to 111.2° for Au/ZnO@Si-c(0.5) (0.5 mL per gram of catalyst), but to 90.1° for Au/ZnO@Si-c(2.0), indicating that a higher amount of TMCS does not further enhance the hydrophobicity.

### 2.9. Catalytic Performance

**Figure 9:**
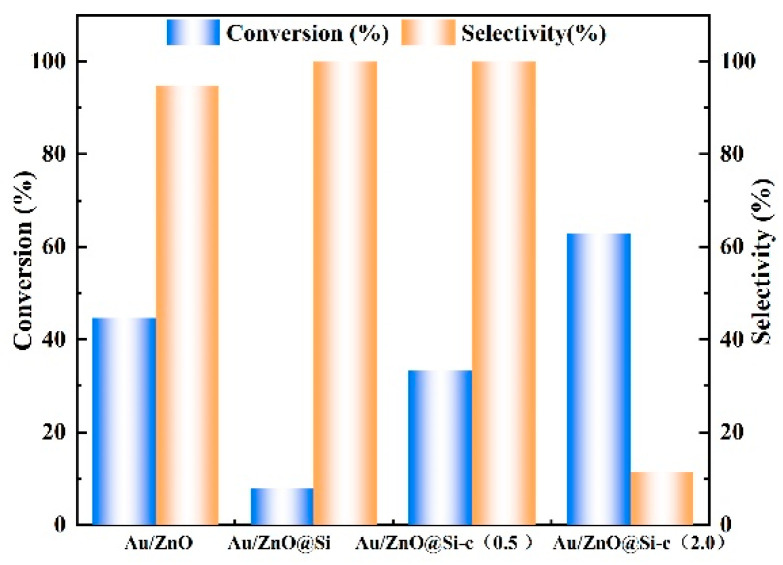
Catalytic performance of catalysts (reaction conditions: methanol and MAL 15 mL, Methanol/MAL ratio (mol/mol) = 20, catalyst 0.5g, 80 °C, and 0.5 MPa O_2_).

The samples were employed for a couple of MAL and methanol using oxygen as an oxidant, with the performance shown in Figure 9. The Au/ZnO presented a high conversion of MAL at 45%, and the selectivity for MMA was 96% after 2 h of the reaction. The good catalytic effect of the Au/ZnO catalyst is due to the small particle size of Au and the uniform distribution of the support. Although the basic sites of the Au/ZnO@Si catalyst increased after the addition of silicon shells, the addition of silicon shell resulted in the enlargement of Au particles and the decrease of the active site of gold, resulting in the decrease of catalyst activity. After hydrophobic modification, the conversion of Au/ZnO@Si-c(0.5) decreased while the selectivity increased, and the conversion of Au/ZnO@Si-c(2.0) increased, but the selectivity was only 12%. As shown in Table S2, it is calculated that the TON value of the catalyst is the highest, which is 1394. These results suggest that careful optimization of the hydrophobicity of the catalyst is necessary for achieving high conversion and selectivity in this reaction. The mechanism of oxidative esterification on catalysts can be described as follows: methanol is adsorbed on the surface of Au nanoparticles, and the alkaline sites of the supporter or alkaline additives promote the breaking of O–H bonds and the removal of β-H, thereby promoting the formation of methoxy groups. The methoxy nucleophilic attack on MAL leads to the formation of intermediate hemiacetal, which removes β-H and forms MMA. On the surface of Au nanoparticles, the β-H that was removed in the previous step is oxidized via oxygen, ultimately forming H_2_O [20]. The hydrophobic groups present in the hydrophobic catalyst play a crucial role in removing the water formed during the reaction from the pores. This prevents the formation of a water film at the active site and promotes the progress of the oxidative esterification reaction in the forward direction.

By employing hydrophobic catalysts, the water generated during the reaction is efficiently removed, allowing the oxidative esterification process to proceed smoothly and enhancing the overall reaction efficiency.

Figure S3 illustrates the effect of varying amounts of TEOS on the catalytic performance of the samples for the oxidative esterification of MAL with methanol. As shown, the addition of 2.5 mL of TEOS to the catalyst preparation led to a decrease in conversion but an increase in selectivity. The conversion of subsequent hydrophobic catalysts and hydrophilic silicon shell catalysts slightly improved despite the decrease in TEOS concentration. These results suggest that the balance between conversion and selectivity in this reaction is highly dependent on the amount of TEOS used in catalyst preparation, as well as the hydrophobicity of the final catalyst.

### 2.10. Kinetics

Kinetics were developed based on the kinetics model established by our group [18]. In the blank experiment conducted without the catalyst, the reaction conditions were as follows: 80 °C, 0.5 MPa O_2_, and n_MeOH_/n_MAL_ = 20. After 120 min of reaction, the conversion of MAL was 25%, but no methyl methacrylate was generated. This suggests that without a catalyst, the active species responsible for the conversion of MAL to MMA is absent. However, when a hydrophobic carrier was prepared by directly constructing a silicon shell and performing hydrophobic treatment on the ZnO support, and this catalyst was used in the reaction, the result was still the absence of MMA as a product. This indicates that the active component responsible for the catalytic conversion of MAL to MMA is Au in the catalyst.

The linear relationship of ln C_MAL_–ln r was investigated to obtain reaction orders at different temperatures, which finally determined the reaction order of oxidative esterification as 1.985. The kinetic equation of the reaction was

(1) $${\;r=k(C}_{\mathrm{MAL}}{)}^{1.985}.$$

**Figure 10:**
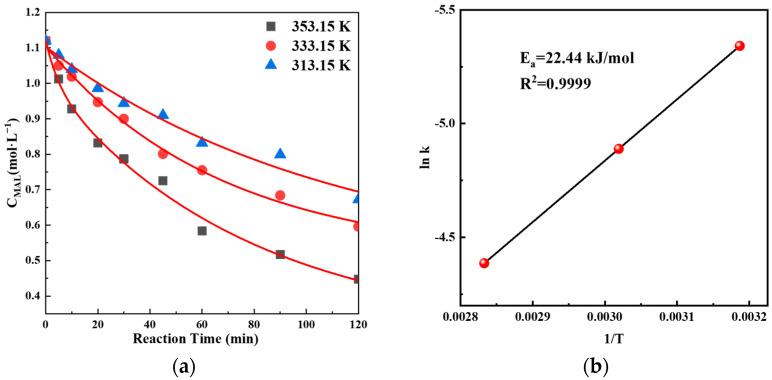
(**a**) Fitting curves for MAL concentration and reaction time at different temperatures. Reaction conditions: catalyst 0.5 g, methanol and MAL (15 mL), Methanol/MAL ratio (mol/mol) = 20, and P(O_2_) = 0.5 Mpa; (**b**) Arrhenius plot of oxidative esterification reaction.

Based on previously determined kinetic models, kinetic studies of oxidative esterification reaction with the catalyst were carried out. Fitting curves for MAL concentration at different reaction temperatures can be seen in Figure 10a. The activation energy of the reaction was studied using the Arrhenius formula. As shown in Figure 10b, the value of activation energy E_a_ was determined as 22.44 kJ mol^−1^.

## 3. Discussion

The Au/ZnO@Si-c catalyst was obtained through hydrophobic modification of the Au/ZnO@Si catalyst using trimethylchlorosilane (TMCS) reagent. The water droplet contact angle of the Au/ZnO@Si catalyst is less than 90°, indicating that it is a hydrophilic catalyst, while the water droplet contact angle of the Au/ZnO@Si-c catalyst is greater than 90°, indicating that a hydrophobic catalyst has been successfully prepared. Compared with the Au/ZnO@Si catalyst, the Au/ZnO@Si-c catalyst exhibits a smaller specific surface area and larger pore size, which may be attributed to the formation of stacked pores during hydrophobic modification. After hydrophobic modification, the gold loading of the Au/ZnO@Si-c catalyst decreased, which may be due to the extended ultrasound time during the catalyst’s hydrophobic modification process, resulting in the loss of some Au. The reduced desorption observed in CO_2_-TPD for the Au/ZnO@Si-c catalyst suggests a decrease in the number of alkaline groups after modification, indicating successful grafting of hydrophobic groups onto the catalyst’s surface. The catalyst prepared using the deposition–precipitation method exhibits an Au particle size of 4.33 nm. However, the Au particle size of the Au/ZnO@Si and Au/ZnO@Si-c catalyst increased. It indicated that the construction of the silicon shell and the hydrophobic modification treatment would affect the aggregation of gold nanoparticles directly loaded on the support. The deposition–precipitation method for preparing the gold catalyst does not provide effective control over the subsequent operations’ impact on the size of Au particles. In our future work, we aim to investigate alternative preparation methods that can effectively control the size of Au particles, thereby reducing the influence of core–shell construction and hydrophobic treatment on the Au particle size.

## 4. Materials and Methods

### 4.1. Materials

Chlorauric acid (HAuCl_4_·4H_2_O), nano Zinc oxide (ZnO, 99.8%, 50 ± 10 nm), Tetraethoxysilane (TEOS, AR), ammonia (25~28%, AR), ethanol (AR), n-hexane (AR) and Chlorotrimethylsilane (TMCS, AR) were obtained from Shanghai Aladdin Biochemical Technology Co., Ltd. (Shanghai, China).

### 4.2. Methods

#### 4.2.1. Synthesis of Hydrophobic Support of ZnO

Using the urea deposition–precipitation method, 0.5 mL of HAuCl_4_·4H_2_O solution (0.1 mol/L) was added to 50 mL of deionized water, and 3.5 g urea was added to the HAuCl_4_·4H_2_O aqueous solution. The resulting solution was heated to 80 °C, and 1 g ZnO was added for 3 h. Then, it was filtered, and the solid samples were washed repeatedly with deionized water until no residual chloride ions were present in the solution. The precipitation was continued to filter, and the resulting sample was subsequently dried in air at 80 °C for 12 h. Finally, it was burned for 2 h in an airflow at 250 °C to obtain the corresponding catalyst.

#### 4.2.2. Synthesis of Au/ZnO@Si

The core–shell Au/ZnO@Si was prepared using the modified Stöber method. Typically, 1.0 g of the prepared Au/ZnO was dispersed in 300 mL of ethanol (AR) via ultrasonication. Then, 2.5 mL of tetraethoxysilane (TEOS, AR) was added. After stirring under 450 rpm for 4 h, 5 mL of ammonia (25–28%, AR) and 20 mL of water were added. The mixture was stirred for another 4 h. Subsequently, the product was washed with ethanol and dried at 100 °C for 11 h. 

The effect of different amounts of TEOS addition was investigated. For convenience, the catalyst prepared by adding 2.5 mL of TEOS was abbreviated as Au/ZnO@Si. The catalyst prepared by adding 1.25 mL of TEOS is abbreviated as Au/ZnO@Si(1/2), and the catalyst prepared by adding 0.625 mL of TEOS is abbreviated as Au/ZnO@Si(1/4).

#### 4.2.3. Synthesis of Hydrophobic Catalysts

To obtain hydrophobic encapsulation, further organic modification was carried out. The previously prepared Au/ZnO@Si catalyst was preheated in a vacuum oven at 150 °C for 11 h. Then, n-hexane and chlorotrimethylsilane (TMCS) were added, with y mL of TMCS per gram of Au/ZnO@Si (where y represents the amounts of TMCS used, which were 0.5 and 2.0 mL per gram). The resulting mixture was ultrasonically treated at room temperature for 3 h. The product was then washed with n-hexane and dried in a vacuum oven at 80 °C for 11 h. 

For convenience, the hydrophobic catalysts prepared with different amounts of TMCS were recorded separately as Au/ZnO@Si-c(0.5) and Au/ZnO@Si-c(2.0).

### 4.3. Characterization

The phase structure of the catalysts was characterized on a Bruker AXS D8 Advance X-ray diffractometer, which diffracted Cu-Kα rays (λ = 1.5406 Å) and scanned the range of 10°–85° at a speed of 4°/min. The adsorption and desorption analysis of N_2_ was completed on the ASAP 2460 surface area analyzer. Before the test, the samples were pretreated at 200 °C for 4 h in a vacuum. The BET equation and BJH method were used to analyze the specific surface and pore size distribution, respectively. TEM images and element mapping measurements were performed under a TECNAI G2 F20 high-resolution transmission electron microscope with a working voltage of 200 kV. More than 100 Au nanoparticles were evaluated to determine each sample’s Au particle size distribution. SEM was performed on the FEI Scanning Electron Microscope Apreo (Dutch PHILIPS XL-30 model). CO_2_-TPD was completed on the AutoChem II chemical adsorption analyzer (McMuratic Instruments Co., LTD, Shanghai, China). The materials were decontaminated with helium at 200 °C, and a mixture of hydrogen (10%) and argon (90%) was used for temperature-programmed reduction. In the TPD process, a mixture of carbon dioxide (10%) and helium (90%) was used to make the catalysts adsorb CO_2_, and high-purity helium was used for desorption. The heating rate was set to 10 °C/min, and the signal from 50 °C to 550 °C was recorded. UV–Vis analysis was performed on the UV-2600 instrument by SHIMADZU (Kyoto, Japan). The slit width was set to 5.0, and the test method was reflectance. XPS was analyzed on the PHI5700 spectrometer using monochromatic Al Kα as the X-ray source. In the data processing process, C1s = 284.8 eV was used to calibrate the charge of the samples. The static contact angles of water drops on the surfaces were measured with an automatic contact angle meter combined with flash camera equipment (Shanghai Sunzren Instrument Co., Ltd., Shanghai, China) at room temperature. The measured contact angles were an average of five measurements.

### 4.4. Catalytic Activity Test

The oxidative esterification reactions were performed in a 50 mL stainless steel autoclave. The mole ratio of methanol and MAL was 20 during preparation. The mixed solution (15 mL) was filled into the steel autoclave with 0.5 g of Au catalyst and 0.02 g of K_2_CO_3_. After charging O_2_ to a pressure of 0.5 MPa, the blending solution was heated to 80 °C, and then the reaction was started with stirring at 300 rpm. After 2 h of reaction time, the reaction was halted by stopping stirring and introducing oxygen. The reactor was quickly cooled down to room temperature. The products were separated using an organic microfilter and then analyzed using an Agilent gas chromatograph comprising an FID detector and a capillary column (PEG-20M, 30 m × 0.25 mm × 0.5 μm) using n-heptane as an internal standard for quantification. Conversion and selectivity were calculated using the following equations:

(2) $$X_{\mathrm{MAL}}(\mathrm{mol})=\frac{\mathrm{mol}\;\mathrm{of}\;\mathrm{MAL}\;\mathrm{reacted}}{\mathrm{mol}\;\mathrm{of}\;\mathrm{MAL}\;\mathrm{initially}}\times \;100\;$$

(3) $$S_{\mathrm{MMA}}(\mathrm{mol})=\frac{\mathrm{mol}\;\mathrm{of}\;\mathrm{MMA}\;\mathrm{generated}}{\mathrm{mol}\;\mathrm{of}\;\mathrm{MAL}\;\mathrm{converted}}\times \;100\;$$

## 5. Conclusions

The present work demonstrates the successful encapsulation of Au/ZnO catalysts with hydrophilic or hydrophobic silicon shells and the impact of such modifications on their catalytic performance for the oxidative esterification of MAL with methanol. The addition of a silicon shell resulted in a decrease in gold active sites and conversion, as well as an increase in selectivity. Hydrophobic modification further improved selectivity but reduced conversion. Finally, the effect of TEOS concentration on catalytic performance was also investigated, revealing a balance between conversion and selectivity that is highly dependent on TEOS concentration and catalyst hydrophobicity. The catalytic performance of hydrophobic catalysts needs to be improved, and further research will be carried out in the future.

## Figures and Tables

**Table 1:** BET surface area and crystalline diameter of samples.

Samples	BET Surface Area (m^2^ g^−1^) ^a^	Pore Size(nm) ^a^	Au Loading (wt%) ^b^
Au/ZnO	43.4	21.6	0.12
Au/ZnO@Si	20.1	14.5	0.10
Au/ZnO@Si-c(0.5)	17.8	20.4	0.04
Au/ZnO@Si-c(2.0)	7.6	16.9	0.04

^a^ The BET surface area and pore size were obtained from nitrogen adsorption and desorption isotherms; ^b^ Calculated using ICP data.

**Table 2:** Desorption of CO_2_ of the catalysts.

Catalysts	Desorption of CO_2_ (mmol·g^−1^) ^a^	
	Low-Temperature Peak ^b^	High-Temperature Peak ^c^
Au/ZnO	-	0.9
Au/ZnO@Si	0.83	2.25
Au/ZnO@Si-c(0.5)	0.41	1.0
Au/ZnO@Si-c(2.0)	0.45	0.49

^a^ The amount of CO_2_ adsorption was calculated via integral calculation. ^b^ Temperature: 50 °C–250 °C. ^c^ Temperature: 250 °C–550 °C.

**Table 3:** XPS analysis of Au 4f for catalysts.

Catalysts	Au^0^		Au^3+^	
	BE (eV)	Fraction (%)	BE (eV)	Fraction (%)
Au/ZnO	83.21	55.50	85.54	44.50
Au/ZnO@Si	83.11	6.77	86.65	93.23
Au/ZnO@Si-c(0.5)	84.10.	49.90	86.70	50.10
Au/ZnO@Si-c(2.0)	83.00	36.47	88.22	63.53

## Data Availability

Data are contained within the article and Supplementary Materials.

## Supplementary Materials

The following supporting information can be downloaded at https://www.mdpi.com/article/10.3390/molecules29081854/s1. Figure S1: EDS diagram of Au/ZnO@Si catalyst; Figure S2: EDS diagram of Au/ZnO@Si-c(2.0) catalyst; Figure S3: Catalytic performance of catalysts: (a): the catalytic performance of catalysts prepared with different amounts of TEOS for the reaction; (b): the catalytic performance of hydrophobic catalysts prepared with the same amount of hydrophobic reagent TMCS under different amounts of TEOS; Table S1: XPS analysis of O1s for catalysts; Table S2: Catalyst performance.
