# Supplementary material for: Preparation of Hydrophobic Au Catalyst and Application in One-Step Oxidative Esterification of Methacrolein to Methyl Methacrylate
Source: Molecules. 2024 Apr 19;29(8):1854. doi: 10.3390/molecules29081854 (PMC11055172; doi:10.3390/molecules29081854)
Supplement: Supplementary file 1 [file molecules-29-01854-s001.zip › molecules-2951018-supplementary.pdf]

Supporting Information

Preparation of Hydrophobic Au Catalyst and Application in One-Step Oxidative Esterification of Methacrolein to Methyl Methacrylate

Yanxia Zheng [1,†], Yubo Yang [1,†], Yixuan Li [2], Lu Cai [1], Xuanjiao Zhao [1], Bing Xue [2,*], Yuchao Li [1,*], Jiutao An [3] and Jialiang Zhang [4]

1 Institute of Clean Chemical Technology, School of Chemistry and Chemical Engineering, Shandong Collegial Engineering Research Center of Novel Rare Earth Catalysis Materials, Shandong University of Technology, Zibo 255049, China; yanxia2020@126.com (Y.Z.)
2 School of Mechanical Engineering, Shandong University of Technology, Zibo 255049, China
3 School of Resources and Environmental Engineering, Shandong University of Technology, Zibo 255049, China
4 Shandong Mingsheng Environmental Protection Technology Co., Ltd., Jinan 250000, China
* Correspondence: xuebing@sdut.edu.cn (B.X.); cyulee@126.com (Y.L.)
† These authors contributed equally to this work.

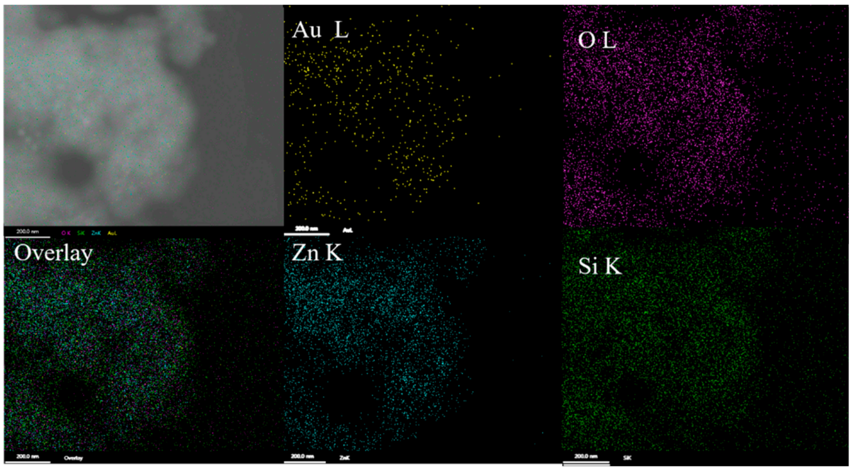

Figure S1. EDS diagram of Au/ZnO@Si catalyst.

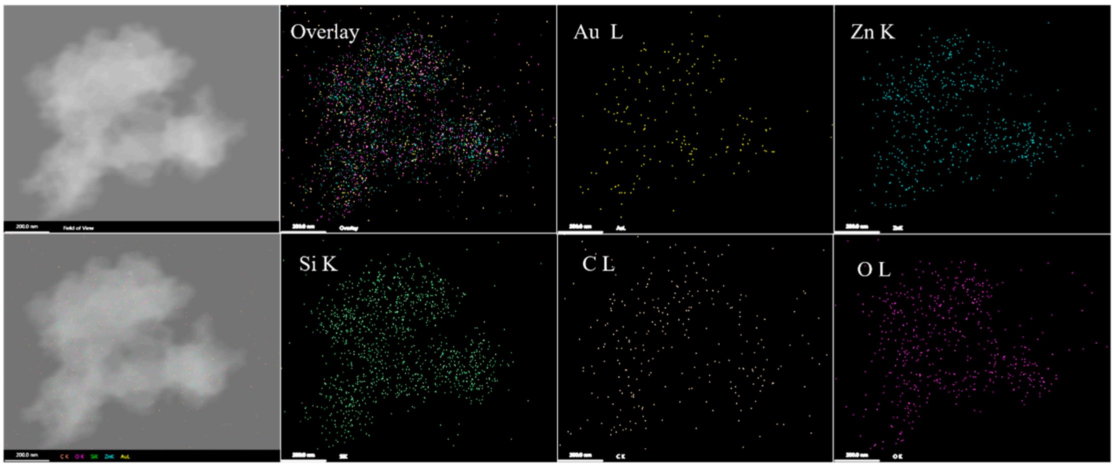

Figure S2. EDS diagram of Au/ZnO@Si-c(2.0) catalyst.

Table S1. XPS analysis of O1s for catalysts.

	O_I		O_{II}		O_{III}	
	BE (eV)	Fraction (%)	BE (eV)	Fraction (%)	BE (eV)	Fraction (%)
Au/ZnO	530.12	44.72	531.32	39.69	532.31	15.59
Au/ZnO@Si	532.15	60.28	533.0	25.18	533.86	14.53
Au/ZnO@Si-c(0.5)	532.33	54.34	533.2	39.09	533.42	6.57
Au/ZnO@Si-c(2.0)	532.86	66.14	533.34	27.71	534.31	6.15

Table S2. Catalyst performance

	Con./%	Sel./%	TON	Yield/%
Au/ZnO	44.6	96	595	42.82
Au/ZnO@Si	17.89	100	302	17.89
Au/ZnO@Si-c(0.5)	33.3	100	1394	33.3
Au/ZnO@Si-c(2.0)	63	12	310	7.12

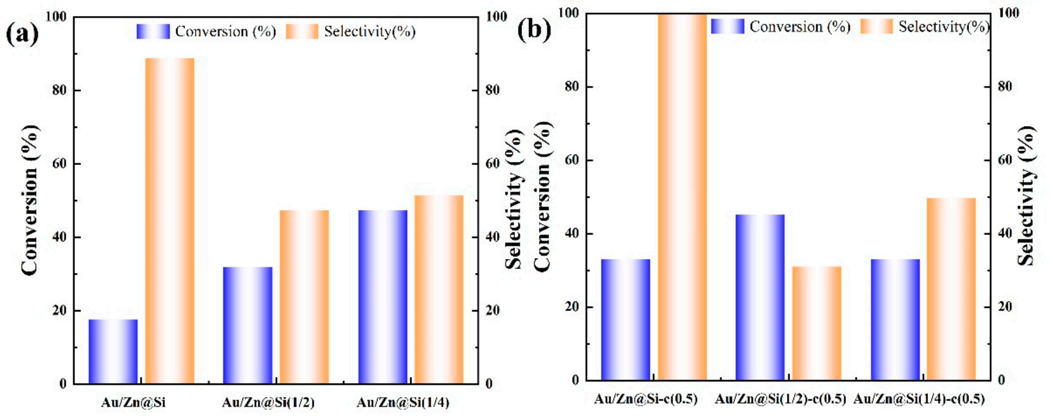

Figure S3. Catalytic performance of catalysts: (a): the catalytic performance of catalysts prepared with different amounts of TEOS for the reaction; (b): the catalytic performance of hydrophobic catalysts prepared with the same amount of hydrophobic reagent TMCS under different amounts of TEOS.
